# Directed evolution of synthetic coexistence: a new path towards ecosystem design?

**DOI:** 10.1093/synbio/ysaa025

**Published:** 2020-11-24

**Authors:** Sonja Billerbeck

In nature, microorganisms never live alone but rather build interconnected communities, able to perform complex biochemical tasks that are essential to the function of most of earth’s ecosystems. By comparison, the microorganisms we rely on as chassis for synthetic biology lead relatively simple, isolated lives. However, mimicking the natural complexity of microbiomes can help synthetic biologists realize more advanced functionalities; e.g. as shown for the efficient biosynthesis of oxygenated taxanes (precursors of the antitumor agent paclitaxel) in a two-species ecosystem (1).

Recently, microbial ecologists demonstrated that it is possible to evolve coexistence between two important synthetic biology chassis in just 100 days, opening the possibility to rapidly assemble synthetic ecosystems by directed evolution (2).

Several challenges stand in the way of designing synthetic ecosystems (3). Assuming that adequate cell-to-cell communication is achieved (4), the question of stable coexistence remains. How can differentially engineered species grow together while competing for the same resources and exhibiting different growth rates? Without stable coexistence, engineered ecosystems will quickly disassemble and lose their ability to fulfil their designed task. Current approaches rely on metabolite cross-feeding, but this requires heavy engineering and poses metabolic burden on each ecosystem member (3).

Recent findings from the field of microbial ecology could provide a powerful alternative to the coexistence challenge. Researchers from Michael McDonald’s laboratory report in Nature’s *ISME Journal* that stable cocultures of *Escherichia coli* and *Saccharomyces cerevisiae* can be established within 1000 generations (100 days) of directed co-evolution in simple microtiter-plate cocultures. Both species compete for the same resources, and *E. coli* grows faster than *S. cerevisiae*. Theory predicts that under such strong competition *E. coli* would drive *S. cerevisiae* extinct. While this happened in 58 out of their 60 replicate cultures, two cultures still contained both species after an initial 420 generations. The authors then further directed the evolution of coexistence by coculturing the coexisting isolates for another 580 generations in 30 replicates. Eventually four cultures developed stable coexistence at a fixed ratio. Impressively, coculture-evolved *S. cerevisiae* isolates were able to re-establish this ratio even when inoculated at low cell numbers into a culture of their co-evolved *E. coli* partner. Ancestral *S. cerevisiae* was not able to do that, showing that the acquired evolutionary changes were necessary and sufficient to coexist with *E. coli.* The *E. coli* partner in return had acquired mutations that enabled it to better access media resources either provided by or not used by its coevolved *S. cerevisiae* partner, showing the start of evolved dependence or occupation of non-competitive niches.

For synthetic biology, these results are important as they indicate that evolution of co-existence and the beginnings of interconnected behaviour happens in a surprisingly short timeframe (∼3.5 months). This opens the door to a number of applications that were previously limited by the coexistence challenges.

For example, the area of agricultural waste valorization where cellulosic material is biosynthetically converted into valuable chemicals would profit from evolved coexistence between cellulolytic bacteria or fungi that extracellularly degrade cellulose into glucose—than acting as the shared resource—and genetically tractable workhorses like *S. cerevisiae*, engineered to produce the chemical of interest.

There are several important next steps that studies bridging synthetic biology and microbial ecology should take to explore this area further: Is the directed evolution of coexistence scalable to multiple species and other species-pairs? How resilient are evolved cocultures to environmental perturbations and the addition of engineered functionalities? Finally, can we extract genetic rules for coexistence in order to rationally engineer ecosystems in the future?

*Conflict of interest statement*. None declared.
